# Plasma with Added Protease Inhibitors Improves Alpha- and Beta-CGRP Measurement Compared to Serum: Towards a Reliable Biomarker for Chronic Migraine

**DOI:** 10.3390/ijms26209958

**Published:** 2025-10-13

**Authors:** Lucía de la Guerra-Sasián, Gabriel Gárate, Jorge Madera, Sara Pérez-Pereda, Marta Pascual-Mato, Vicente González-Quintanilla, Julio Pascual, María Muñoz-San Martín

**Affiliations:** Instituto de Investigación Marqués de Valdecilla (IDIVAL), Hospital Universitario Marqués de Valdecilla & Universidad de Cantabria, 39011 Santander, Spain

**Keywords:** CGRP, migraine, protease inhibitors, biomarker, plasma, serum

## Abstract

The neuropeptide calcitonin gene-related peptide (CGRP), especially α-CGRP, is central in migraine pathophysiology. Although CGRP is a therapeutic target and potential biomarker, inconsistencies in measurement procedures need to be further studied for reliable results. This study aims to analyze factors influencing plasma CGRP measurement. Chronic migraine (CM) patients were recruited in our Headache Unit. Blood samples were collected before and during treatment with CGRP monoclonal antibodies, processed and stored. Levels of CGRP were measured with isoform-specific enzyme-linked immunosorbent assay (ELISA) tests. Statistical tests were used to assess concentration changes and group differences. The addition of protease inhibitors (PIs) to plasma samples significantly increased α-CGRP level detection, with a smaller effect on β-CGRP. No correlation was found between the α- and β-CGRP levels in plasma. The plasma-PI samples showed higher CGRP concentrations than in serum. The α-CGRP levels decreased during treatment while the β-CGRP levels remained stable. α-CGRP and age correlated negatively, but no sex-related differences were observed either for α- or β-CGRP. PI improved CGRP detection in plasma. The α-CGRP levels, which were influenced by age, decreased with specific treatment, suggesting its potential role as a biomarker. In contrast, β-CGRP remained stable, suggesting independent regulation of both isoforms.

## 1. Introduction

Migraine is a highly prevalent and disabling neurological disorder that involves headache attacks as well as nausea, photophobia or phonophobia among other symptoms. This disorder continues to be the second leading cause of neurological disability worldwide [1,2], and the first among young women according to the Global Burden Disease of 2019; in fact, its prevalence increased substantially among young adults and youths between 1990 and 2021 as explained in the Global Burden Disease of 2021 [3].

Its incidence is significantly higher in women than men, occurring 3–4 times more often in adult females than in males [4]. The tendency decreases after menopause, suggesting that changes in ovarian hormones might play a key role in its pathophysiology. In fact, several studies relate hormonal changes as a trigger in developing a migraine attack [5,6]. For instance, chronic migraine (CM) (headaches occurring 15 or more days/month [7] for 3 or more months) has been reported to have a prevalence of 2.5 to 6.5 times higher (1.7–4%) in females than in males (0.6–0.7%) [7,8].

To date, the pathophysiology of migraine remains poorly understood [2]. Nevertheless, it is well established that migraine has a strong genetic component. Family and twin studies have detected 30–60% heritability of migraine, and recent genome-wide association studies have identified target genes of the calcitonin gene-related protein (CGRP) pathway [9,10]. In addition, it has also been described that trigeminal vascular system activation is required for the headache to develop [11]. This activation leads to the release of vasoactive neuropeptides such as CGRP. This peptide has been highly associated with migraine for years [12], since it was the only peptide consistently elevated within a migraine attack in a study conducted in 1990 [13,14].

CGRP is a multifunctional neuropeptide of 37 amino acids with several functions that results from an alternative splicing of the calcitonin gene transcript (*CALCA*), which leads to the alpha-CGRP (α-CGRP) isoform, whereas a different gene (*CALCB*) encodes the beta-CGRP (β-CGRP) isoform of the peptide [15,16,17,18,19]. Both isoforms are distributed throughout the body and differ in only 3 of the 37 amino acids that compose them [20]. α-CGRP has been described to be predominant in the nervous system (both central and peripheral) and highly abundant in pericerebral vessels as well as in the trigeminal ganglion (TG) [21], whereas β-CGRP is higher in the enteric nervous system and linked with the gastrointestinal effects of CGRP in humans [17,22,23,24].

Animal studies have demonstrated that CGRP is exclusively found in the cytoplasm of Purkinje cell bodies [25], and, although both isoforms of CGRP are found in the neuronal cell bodies of TG neurons, only α-CGRP is localized within TG axons [26]. In both humans and rats, CGRP and its receptor (CGRP-R) components are differentially localized: CGRP in small neurons and unmyelinated C-fibers, and CGRP-R components in larger neurons and myelinated A-fibers. This anatomical segregation might highlight the complexity of CGRP signaling in the CNS, supporting its pivotal role in migraine pathophysiology [27,28,29].

Because of these findings, several CGRP-based drugs, such as the generation of monoclonal antibodies (mAbs) against CGRP ligand or CGRP-R, have been developed. Four monoclonal antibodies have been approved for migraine prevention (erenumab, eptinezumab, fremanezumab, galcanezumab) [15,30], the first of them targeting the CGRP-R and the three others targeting the CGRP ligand. Recently, small oral molecules called “gepants” [31] have emerged to complement mAbs as they also block the CGRP receptor.

The importance of CGRP extends beyond its use as a possible therapeutic target, as it has also been proposed as a biomarker for migraine and for its therapeutical response [32,33,34], particularly α-CGRP due to its predominance in both the central and peripheral nervous system, as mentioned before. This is supported by a study in which treatment with mAbs progressively restored α-CGRP levels by 3 months, and the data obtained supported a role of this neuropeptide as the first dynamic CM biomarker [32,34,35]. Other studies also reflect how mAbs or onabotulinumtoxin A change CGRP levels [33,36,37]. Not only has it been highlighted as a biomarker for migraine, in fact, it has also been proposed for early detection and tracking the disease progression of various neurodegenerative diseases (NDDs) [38]. Regarding β-CGRP, it has also been recently shown that its levels are elevated in COVID-19 patients experiencing diarrhea [15,39], whereas they are decreased across all subtypes of inflammatory bowel disease [40].

Although CGRP levels tend to be elevated in migraine, about one-third of migraine patients have CGRP levels similar to subjects without headache [21]. In addition, the significance of blood measurements remains unclear due to the contradictory results among the different studies that have been published and the variability in biological fluids that have been used besides blood, like saliva, cerebrospinal fluid or tears [33,41]. All these impede to confirm whether the elevation of this peptide can be consistently detected or used as a reliable biomarker for migraine [42,43]. The reason for the discrepancies is most probably multifactorial [44], as they may be influenced by methodological and individual parameters such as hormonal changes due to the menstrual cycle, or other comorbidities that are not usually considered as they should.

Therefore, the aim of this study was to determine a more suitable approach to measure CGRP levels, both α- and β-CGRP isoforms, in plasma samples from CM patients. Moreover, the effect of several aspects including the addition of protease inhibitors (PIs); measurements in different biological fluids (plasma or serum); the use of treatments or sex and age differences in CGRP levels was also analyzed to delve into the potential of this peptide to be used as the biomarker that it has been proposed to be.

## 2. Results

### 2.1. Effect of the Use of Protease Inhibitors in Plasma

To evaluate whether PI addition might affect the levels and stability of both CGRP isoforms and should be implemented as a routine, plasma samples were processed with and without PI and CGRP levels were determined.

**Figure 1:**
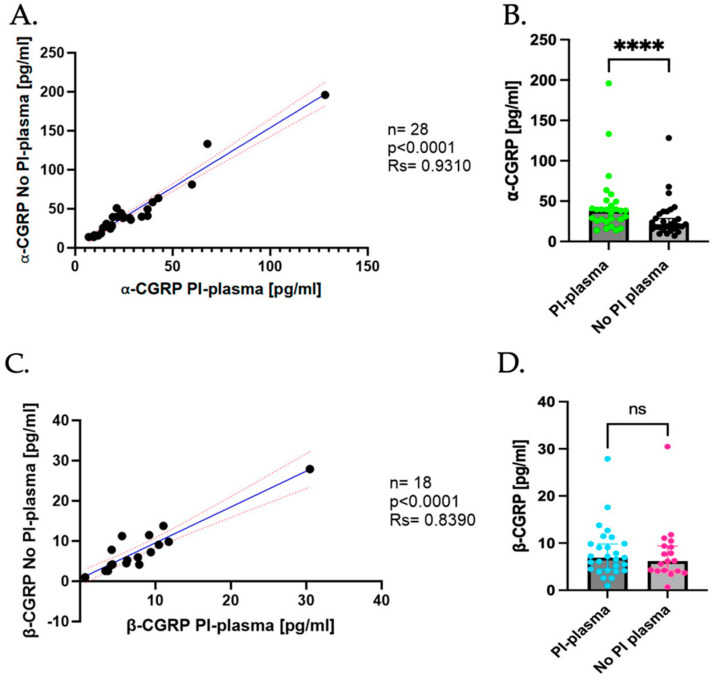
Differences in CGRP levels in plasma with and without PI. (**A**) Correlation of α-CGRP levels in PI-plasma and No PI-plasma. Dark blue line represents the linear regression, and the red dotted line represents the 95% confidence interval (CI). (**B**) Difference in α-CGRP levels in PI-plasma (represented in green) vs. No PI-plasma (represented in black); data are shown as median with CI 95%. Comparison was made using the Wilcoxon Signed-Rank test (PI-plasma median = 38.28 pg/mL, No PI-plasma median = 21.26 pg/mL, *p* ≤ 0.0001). (**C**) Correlation of β-CGRP levels in PI-plasma and No PI-plasma. Dark blue line represents the linear regression, and the red dotted line represents the CI. (**D**) Difference in β-CGRP levels in PI-plasma (represented in light blue) vs. No PI-plasma (represented in pink); data are shown as median with CI 95%. Comparison was made using the Wilcoxon Signed-Rank test (PI-plasma median = 6.909 pg/mL, No PI-plasma median = 6.200 pg/mL, *p* = ns (non-significant)). **** *p* < 0.0001.

A strong positive correlation was observed between the levels of α-CGRP in plasma samples with PI (PI-plasma) and plasma samples without PI (No PI-plasma) (Spearman’s correlation coefficient (rs) = 0.9310, *p* < 0.0001; Figure 1A). The median plasma levels of α-CGRP were significantly higher in the PI-plasma compared to No PI-plasma samples (PI-plasma = 38.29 pg/mL, No PI-plasma = 21.26 pg/mL, *p* < 0.0001; Figure 1B).

Concerning β-CGRP, a positive correlation was also found between the PI-plasma and No PI-plasma samples (rs = 0.8390, *p* < 0.0001; Figure 1C). Nevertheless, no significant difference was detected in the β-CGRP plasma concentrations when comparing the samples treated with PI to those untreated (PI-plasma = 6.909 pg/mL, No PI-plasma = 6.200 pg/mL, *p* > 0.050; Figure 1D).

In summary, these results indicate that the addition of PI significantly increases the detection of α-CGRP in plasma, while it does not affect the β-CGRP levels. This suggests that PI addition could be considered as a useful strategy for more accurate quantification of α-CGRP in plasma samples.

### 2.2. Correlation of α- and β-CGRP Levels

In previously published studies, our group reported that the serum levels of α- and β-CGRP do not correlate in the serum samples of patients. To determine whether this finding is consistent in plasma samples, we performed correlation analyses between both isoforms.

**Figure 2:**
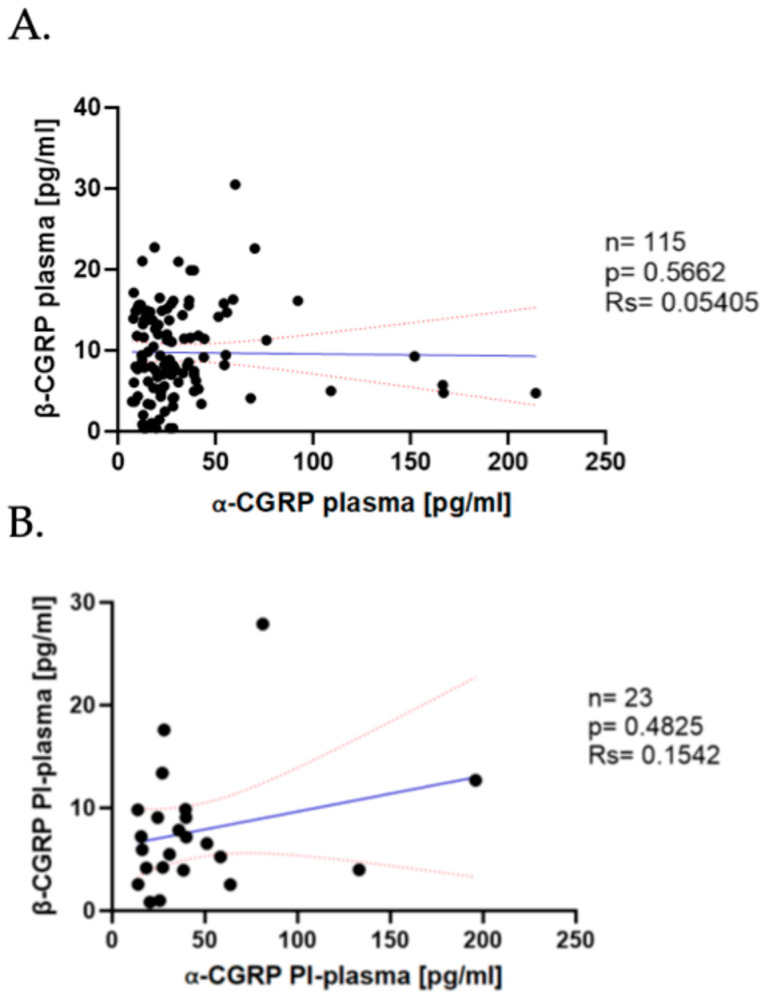
Correlation of α-CGRP and β-CGRP in plasma samples. (**A**) Correlation of α-CGRP levels vs. β-CGRP plasma levels in No PI-plasma. Dark blue line represents the linear regression, and the red dotted line represents the CI. (**B**) Correlation of α-CGRP levels vs. β-CGRP levels in PI-plasma. Dark blue line represents the linear regression, and the red dotted line represents the CI.

As shown in Figure 2, no correlation was found between the No PI-Plasma levels of α-CGRP and β-CGRP in the study cohort (*n* = 115; rs = 0.05405, *p* = 0.05662; Figure 2A). Despite the reduced availability of PI-plasma, when comparing α- and β-CGRP, we observed the same outcome, since no significant correlation was observed between both isoforms (*n* = 23; rs = 0.1542, *p* = 0.4825; Figure 2B).

We can conclude that the lack of correlation between the α- and β-CGRP concentrations already observed in serum was maintained in plasma, no matter whether we used PI in the processing of samples, suggesting that both isoforms of the peptide may be independently regulated in migraine.

### 2.3. Differences in Serum and Plasma Levels of α-CGRP

Since the main biological fluids used to measure the levels of CGRP are plasma and serum, and our group has specialized in the study of this peptide in the latter, we compared measurements in both sample types from patients with CM to determine the correlation between both biofluids.

**Figure 3:**
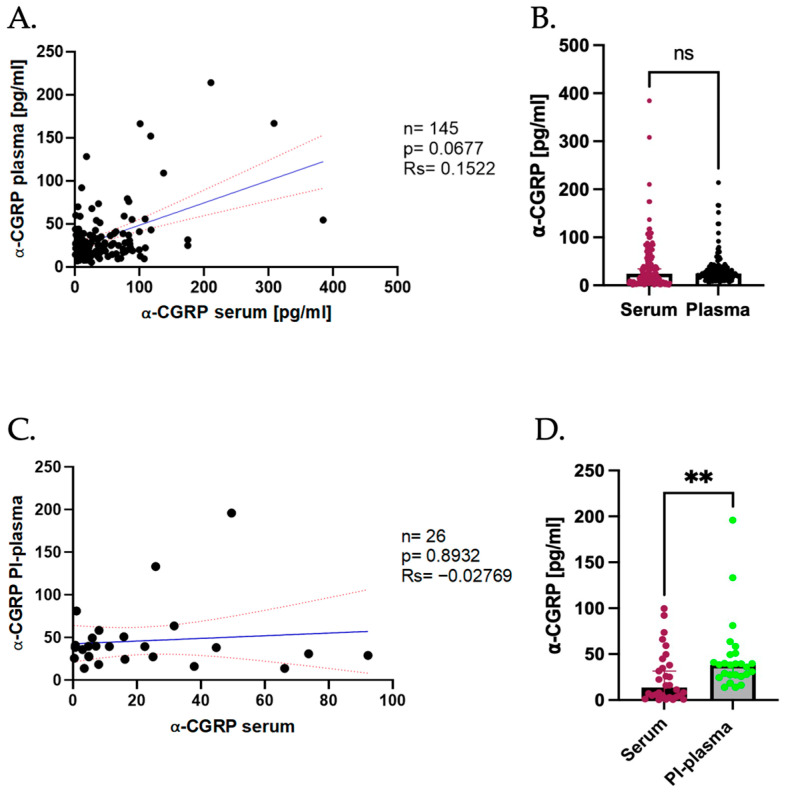
Comparison of α-CGRP levels in serum and plasma. (**A**) Correlation of α-CGRP serum vs. No PI-plasma levels. Dark blue line represents the linear regression, and the red dotted line represents the CI. (**B**) Difference in α-CGRP levels in serum (represented in burgundy) vs. No PI-plasma (represented in black); data are shown as median with CI 95%. Comparison was made using the Wilcoxon Signed-Rank test (serum median = 24.32 pg/mL, No PI-plasma median = 24.94 pg/mL, *p* = ns). (**C**) Correlation of α-CGRP serum levels vs. PI-plasma levels. Dark blue line represents the linear regression, and the red dotted line represents the CI. (**D**) Difference in α-CGRP levels in serum (represented in burgundy) vs. PI-plasma (represented in green); data are shown as median with CI 95%. Comparison was made using the Wilcoxon Signed-Rank test (serum median = 13.63 pg/mL, PI-plasma median = 38.29 pg/mL, *p* ≤ 0.01). ** *p* < 0.010.

A slight positive tendency was observed between the serum and No PI-Plasma levels of α-CGRP (rs = 0.1522, *p* = 0.0677); nevertheless, this correlation was not significant (Figure 3A), indicating that a clear relationship of this isoform in both fluids cannot be established. Comparison of the median levels showed no significant difference between serum and plasma (serum = 24.32 pg/mL; No-PI plasma = 24.94 pg/mL, *p* > 0.05; Figure 3B).

Since the addition of PI appeared to significantly affect the peptide concentration of this isoform, we subsequently performed a similar analysis comparing the serum levels with PI-Plasma. As in No PI-Plasma, in this smaller cohort of patients, no significant correlation was found between α-CGRP in serum and PI-plasma (rs = −0.02769, *p* = 0.8932; Figure 3C). Interestingly, despite the lack of correlation, a significant difference was observed between the median levels in serum and PI-plasma with PI-plasma showing higher concentrations of the peptide (serum = 13.63 pg/mL, PI-Plasma = 38.29 pg/mL, *p* < 0.050; Figure 3D).

Overall, these results indicate that although the α-CGRP levels in serum and plasma are not significantly correlated, the absolute concentrations can differ depending on sample processing. In standard conditions, the plasma levels were slightly higher than the serum levels but not significantly so. However, when PI was added, the α-CGRP plasma levels were significantly elevated compared to serum, suggesting that peptide degradation may influence measurement outcomes.

### 2.4. Differences in Serum and Plasma Levels of β-CGRP

**Figure 4:**
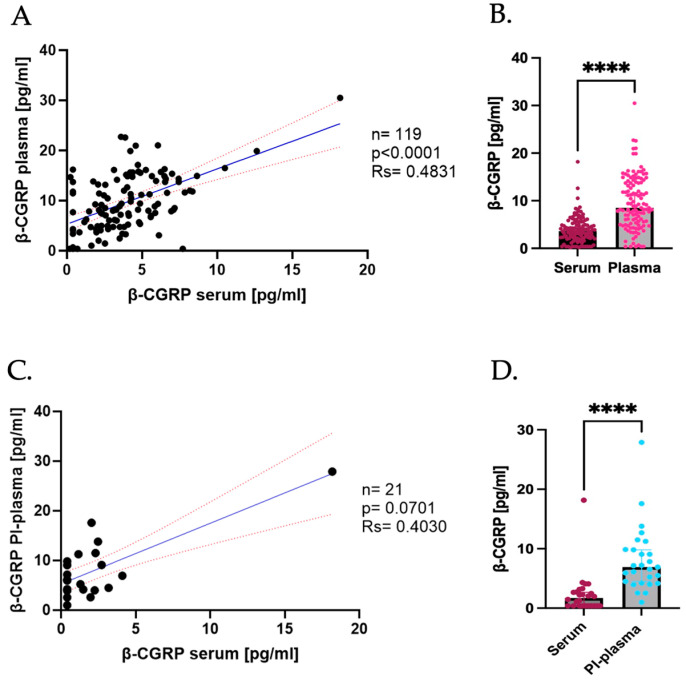
Comparison of β-CGRP levels in serum and plasma. (**A**) Correlation of β-CGRP serum vs. No PI-plasma levels; the dark blue line represents the linear regression, and the red dotted line represents the CI. (**B**) Difference in β-CGRP concentrations in serum (represented in burgundy) vs. plasma (represented in pink); data are shown as median with CI 95%. Comparisons were made using the Wilcoxon Signed-Rank test (serum median = 3.625 pg/mL, plasma median = 8.516 pg/mL, *p* ≤ 0.0001). (**C**) Correlation of β-CGRP serum vs. No PI-plasma levels when using PI; the dark blue line represents the linear regression, and the red dotted line represents the CI. (**D**) Difference in β-CGRP concentrations in serum (represented in burgundy) vs. No PI-plasma with PI (represented in blue); data are shown as median with CI 95%. Comparisons were made using the Wilcoxon Signed-Rank test (serum median = 1.716 pg/mL, plasma with PI median = 6.909 pg/mL, *p* ≤ 0.0001). **** *p* < 0.0001.

As previously performed with α-CGRP, the β-CGRP levels in serum and plasma were compared. A significant positive correlation between the serum and No PI-plasma levels of the β isoform was observed (rs = 0.4831, *p* < 0.0001; Figure 4A), indicating that the β-CGRP levels are strongly associated across both fluids.

Despite this positive correlation and, in contrast with the α-CGRP findings, significantly higher levels in the plasma samples were found and the mean β-CGRP concentration in No PI-plasma was noticeably elevated relative to that in serum (serum = 3.625 pg/mL; No PI-plasma = 8.516 pg/mL, *p* < 0.0001; Figure 4B).

In the PI-plasma samples, a positive tendency in the correlation between serum and PI-plasma was observed; however, this had no statistical significance (rs = 0.4030, *p* = 0.0701; Figure 4C), a result that can be an outcome of the limited sample size or because of the variability in protease activity among individuals. Comparison of the median concentrations revealed that the β-CGRP levels were again significantly higher in PI-plasma than in serum (serum = 1.716 pg/mL, PI-plasma = 6.909 pg/mL, *p* < 0.0001; Figure 4D).

Collectively, these data indicate that plasma consistently yields higher β-CGRP levels than serum, in contrast to the findings for α-CGRP. This suggests that plasma biofluid might provide better sensitivity for β-CGRP detection. In addition, the use of PI may enhance peptide stability, particularly in studies requiring accurate quantification of β-CGRP levels.

### 2.5. Dynamics of CGRP During Treatment

Patients enrolled in our study were prescribed with mAbs anti-CGRP or anti-CGRP-R. CGRP levels were determined at different time points to establish CGRP level dynamics alongside treatment.

We studied both isoforms of CGRP independently in the No PI-plasma samples. Patients were stratified based on the availability of samples at different time points; those with samples collected at baseline (M0) and after 6 months of the treatment (M6); and those for whom samples were also available after 12 months (M12).

**Figure 5:**
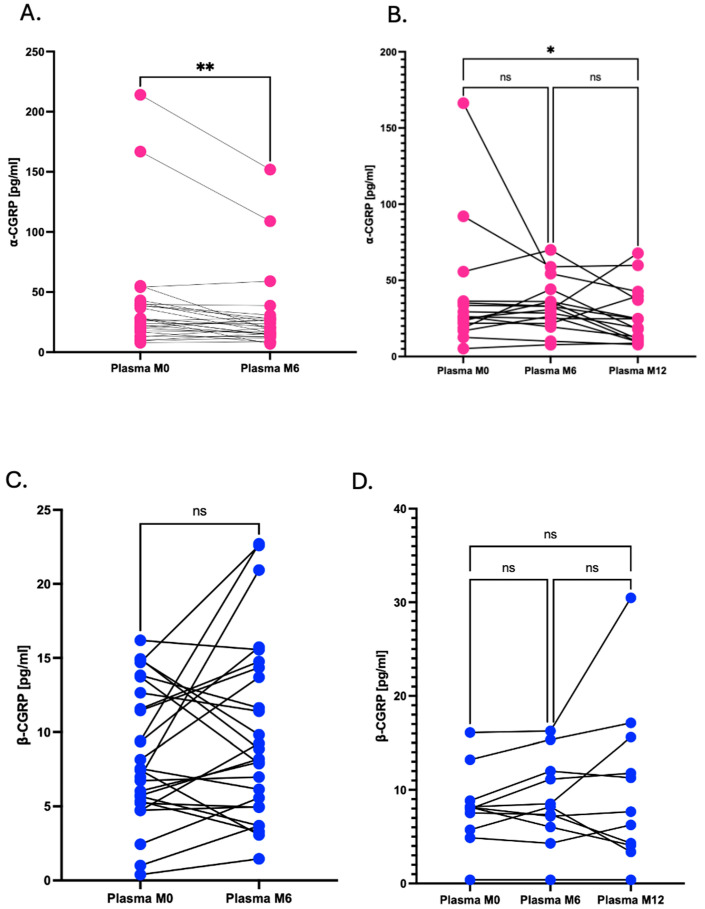
Dynamics of CGRP levels along migraine treatment. (**A**) Changes in α-CGRP concentrations in No PI-plasma before starting the treatment (M0), 6 months after starting treatment (M6)*;* Wilcoxon Signed-Rank test (*p* < 0.01) and (**B**) after 12 months of the beginning of the treatment (M12); Friedman test followed by Dunn’s test. (**C**) Changes in β-CGRP levels in No PI-plasma at M0 and M6 after starting treatment; Wilcoxon Signed-Rank test (ns) and (**D**) M12 Friedman test followed by Dunn’s test. * *p* < 0.050, ** *p* < 0.010.

For the first group of subjects (*n* = 27), we compared plasma α-CGRP at M0 and at M6. Statistical analysis using the Wilcoxon Signed-Rank test indicated that there was a significant change in the α-CGRP concentrations between these two time points (*p* < 0.01), with a higher predominance of negative rank sums, supporting a decreasing tendency in α-CGRP levels post-treatment initiation (Figure 5A).

In the group of patients with samples available at M0, M6 and M12 (*n* = 16), we observed a similar trend in which the α-CGRP levels tended to decrease at M6 and even more at M12. The Friedman test followed by Dunn’s multiple comparisons test revealed significant differences among the three time points, with a significant reduction especially between M0 and M12 (*p* < 0.05, Figure 5B).

Regarding β-CGRP, we also compared the concentrations at M0 and at M6 in the first group of patients (*n* = 26). Even though we observed variability between subjects, we could not observe significant differences between the M0 and M6 time points, as supported by the non-significant result of the Wilcoxon Signed-Rank test (*p* = ns, Figure 5C).

For the group of patients with M0, M6 and M12 samples available (*n* = 11), although some patients showed increases or decreases in the concentration of β-CGRP, the Friedman test followed by Dunn’s multiple comparisons test did not show statistically significant differences among any of the compared time points (*p* = ns, Figure 5D). All results together show that the α-CGRP levels significantly decreased during migraine treatment whilst the β-CGRP levels do not show statistical changes with migraine treatment. This finding might support a potential role as a biomarker for therapeutic response for the α-CGRP isoform.

### 2.6. CGRP Levels Depending on Demographic Variables

Migraine is characterized by its high prevalence among women and its broad age distribution. To investigate the potential impact of these demographic factors on CGRP levels, the plasma samples were analyzed according to patients’ age and sex.

**Figure 6:**
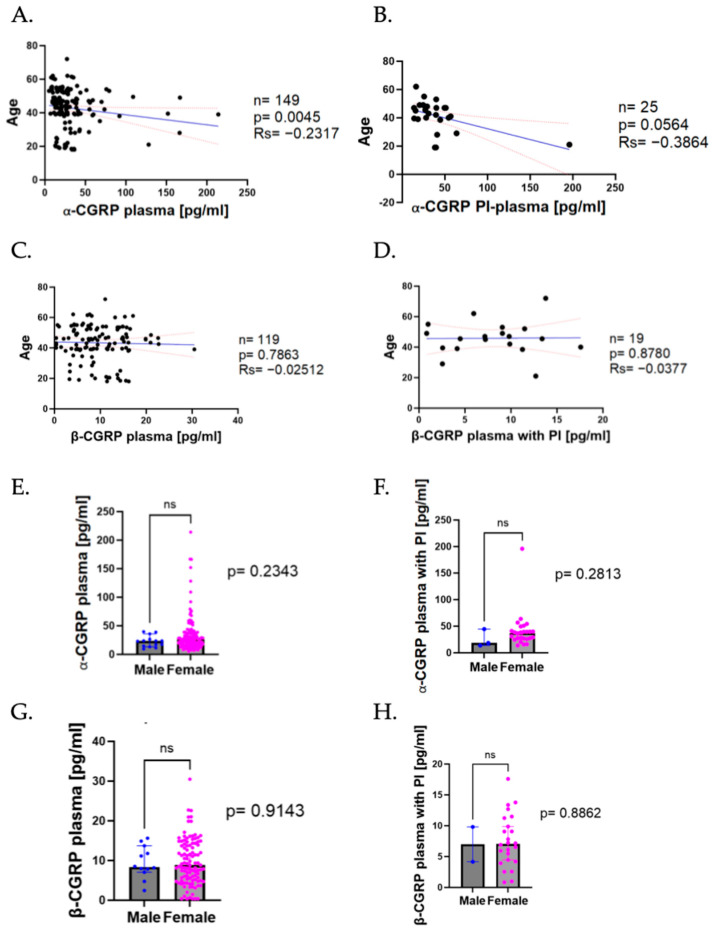
Analysis of CGRP levels in plasma depending on demographic variables. (**A**) Correlation of α-CGRP No PI-plasma levels and age. Dark blue line represents the linear regression, and the red dotted line represents the CI. (**B**) Correlation of α-CGRP PI-plasma levels and age. Dark blue line represents the linear regression, and the red dotted line represents the CI. (**C**) Correlation of β-CGRP No PI-plasma levels and age. Dark blue line represents the linear regression, and the red dotted line represents the CI. (**D**) Correlation of β-CGRP PI-plasma levels and age. Dark blue line represents the linear regression, and the red dotted line represents the CI. (**E**) Comparison of α-CGRP No PI-plasma levels from patients sorted by sex (column A median = 23.31 pg/mL; column B median = 26.09 pg/mL; *p* = ns). (**F**) Comparison of α-CGRP PI-plasma levels from patients sorted by sex. (**G**) Comparison of β-CGRP No PI-plasma levels by sex (column A median = 8.350 pg/mL; column B median = 8.849 pg/mL; *p* = ns). (**H**) Comparison of β-CGRP PI-plasma levels sorted by sex. Data are shown as median with CI 95%. The comparisons in (**E**–**H**) were made using Mann–Whitney U test.

We observed a significant negative correlation between the α-CGRP No PI-plasma levels and age, where the levels of the peptide tended to go downwards as age increased (*n* = 149, rs = −0.2317, *p* = 0.0045; Figure 6A). In PI-plasma, we could not observe that clear correlation, but the α-CGRP levels also presented a light decrease as age increased (rs = −0.3864, *p* = 0.0564, Figure 6B).

On the other hand, for β-CGRP, we did not notice a significant correlation between the peptide levels in No PI-plasma and age (rs = −0.02512, *p* = 0.7963, Figure 6C) nor could we appreciate a significant correlation of PI-plasma with age (rs = −0.03777, *p* = 0.8780, Figure 6D).

Afterwards, the plasma levels were compared depending on patients’ sex. When comparing between men and women, we could not observe statistical differences in our group of patients, neither for the No PI-Plasma (men = 23.31 pg/mL; women = 26.09 pg/mL; *p* = ns; Figure 6E) nor for the PI-plasma samples (men median = 18.35 pg/mL; women median = 37.07 pg/mL; *p* = ns, Figure 6F).

Comparing the β-CGRP plasma levels, we observed similar results, with no significant differences in plasma levels (men = 8.350 pg/mL; women = 8.849 pg/mL; *p* = ns; Figure 6G) or when comparing the PI-treated plasma samples (men = 6.980 pg/mL; women = 7.034 pg/mL; *p* = ns, Figure 6H).

## 3. Discussion

Based on the results obtained in our study, we can draw several conclusions. PI addition to the plasma samples did improve CGRP detection, as it significantly increased the levels of α-CGRP, even if it did not improve β-CGRP isoform detection that much. PIs have already been proposed as an important step regarding sample processing, since proteases and peptidases present in blood such as ECE-1 or IDE have been studied for their possible role in degrading CGRP [45,46]. With a half-life of about 7–10 min, after blood extraction, only a fraction of the original concentration present in the cubital vein will be left, which may alter its original concentration, which is one of the main challenges that could be found when determining the peptide [47]. In spite of this aspect, it is also worth mentioning that if we compare CGRP with other peptides involved in migraine pathophysiology, such as vasoactive intestinal peptide (VIP) or pituitary adenylate cyclase-activating polypeptide (PACAP) [48,49], CGRP has a relatively long half-life, which might account for its prolonged action in migraine [50]. Our results might suggest that CGRP degradation in untreated plasma could lead to an underestimation of the true levels of α-CGRP, which supports and encourages the addition of PI during the processing of plasma to improve CGRP stability and obtain an accurate quantification of this neuropeptide.

As has been performed in previous papers, we also compared both CGRP isoforms in the same individual in plasma. No significant correlation was found between the plasma α- and β-CGRP levels (either with or without PI), which supports the idea that both isoforms may be independently regulated in this disease context. A possible explanation might be that both isoforms of the peptide could be subject to different release mechanisms or to differences in CGRP stability. This might be supported by recent studies in serum that also reported no correlation between both isoforms, meaning that measuring α- or β-CGRP is not interchangeable and could lead to opposite conclusions, given their potentially different behaviors in the same disorder [42]. Studies such as Haanes et al. [26] indicate that the trigeminal system primarily stores and releases α-CGRP, while β-CGRP mainly appeared to reside in the neuronal cell bodies and not in the axons; it is more present in peripheral tissues, which supports the hypothesis that β-CGRP could originate from enteric sources. These observations might lead to further research incorporating functional assays or studies at the cellular level to determine the specific role of each isoform in CM and how this independent regulation could affect disease progression and therapy response.

Regarding the differences between serum and plasma, the α-CGRP levels were not significantly different comparing serum with plasma but the addition of PI in plasma significantly increased its levels, implying that the peptide degradation in plasma could account for lower levels. In contrast, the β-CGRP concentrations were consistently higher in plasma than in serum, no matter whether the plasma had PI added or not. Because of these results, measuring the β-CGRP levels could be performed better in plasma rather than in serum since the levels detected are consistently higher in the first biological fluid.

We also studied the changes in CGRP levels during CM treatment and obtained varied results. The α-CGRP concentrations decreased over the course time of migraine treatment, which might support its potential utility as a biomarker for treatment response in CM. However, the β-CGRP levels did not change significantly during treatment, which could imply different regulatory mechanisms or roles of both isoforms of the peptide in migraine’s pathology. The treatment effect has previously been tested in serum, and similar effects were shown, as the α-CGRP levels were lower three months after the first dose of treatment but the same could not be said for the β-CGRP levels, as no significant difference was observed, which again supports the need for the further investigation of the involvement of each of the CGRP isoforms in migraine [34]. According to a published study, the treatment response in patients with CM is not as influenced by the CGRP levels at baseline as in patients with episodic migraine, which leads us to believe that there must be other biological or genetic components involved [33]. In other studies, it has been observed that migraineurs’ CGRP levels are increased in peripheral circulation outside of attacks, suggesting a possible abnormality of the release from the sensory neurons, which might be related to the higher susceptibility of patients to develop a migraine attack than non-migraineurs [51]. Our results in plasma support and highlight the potential of α-CGRP as a biomarker for treatment response in CM. To confirm this, further studies including responders and non-responders should be performed.

The influence of demographic factors was also studied as several articles had mentioned its importance for developing the disease [52,53]. Apart from CGRP, there are other peptides that have been studied to be influenced by age and sex such as the neuropeptide Y, which is also elevated in unhealthy obesity [54,55], or the neurofilament light chain, which should be adjusted by age when studying its levels [56]. The α-CGRP plasma levels showed a significant negative correlation with age, indicating reduced peptide levels in older patients. This tendency also persisted with the PI-treated plasma samples although it was less pronounced. In contrast, the β-CGRP levels did not show a significant relationship with age, highlighting that both isoforms do not correlate. With these findings, age needs to be considered when analyzing the CGRP levels as a possible biomarker and the values should be adjusted. Regarding sex differences, no significant variation was observed for either the α- or β-CGRP concentrations no matter whether PI was added or not.

Despite the outcomes of our study, several limitations should be considered. The sample size for sex distribution was small, and we did not have enough men to obtain a significant result. Another limitation might be that we did not measure serum and plasma at the same time, since the serum samples of our patients had already been measured for previous studies, so ELISA inter-plate variability may have influenced the results obtained. It should also be considered that the specificity of the ELISA kits used for our work could influence our results, since both isoforms of the peptide are very similar to each other. In fact, it has been postulated that the kit used for the detection of β-CGRP may not be suitable for detecting mature bioactive CGRP in clinical samples, a factor to be taken into consideration in subsequent studies [57]. Finally, blood was drawn from the cubital vein due to its accessibility, although jugular sampling may better reflect local neuropeptide changes. While early studies only found CGRP elevations in the jugular vein [13], subsequent research also demonstrated elevated CGRP in cubital vein samples [35,51,58], not only during migraine attacks but also in the interictal phase [59], and in other biological fluids such as tears [60], saliva [61] or cerebrospinal fluid [62]. Moreover, comparative studies found no significant differences in CGRP or other peptide levels between the jugular vein or cubital fossa samples [63,64].

As for future work perspectives, larger sex-balanced cohorts should be selected to obtain significant results regarding these clinical aspects; since age tended to reduce α-CGRP levels, it could also be an important aspect to consider for treatment prescription depending on the age of the patient. We also suggest that a study including patients that are known to be responders to treatment and those who are also known to be non-responders could be included in a stratified study in order to measure the real use of CGRP as a therapeutic response biomarker. Apart from that, as it has been studied that CGRP could also be a biomarker for NDDs [38], a study comparing the levels of CGRP of patients of both migraine and different NDDs could be performed to determine the specificity of CGRP in CM.

## 4. Materials and Methods

### 4.1. Recruitment of Study Participants

Patients included in this study were recruited from the Headache Unit at the University Hospital Marqués de Valdecilla. In order to be included in the study, subjects had to be older than 17 years old and meet the criteria for mAbs anti-CGRP/CGRP-R treatment prescription in our country (meeting CM criteria after the use of at least three migraine preventatives, including onabotulinumtoxin type A).

Regarding the exclusion criteria, those who were pregnant or breast-feeding women, had any psychiatric disease, were alcohol-dependent individuals or were taking daily medication for other medical reasons were left out of the study.

The study was approved by the Ethics Committee of Investigations with Medications of Cantabria, and its approval was published in the record 28/2020 of 11 December. All participants gave written informed consent for their inclusion in the study [34].

### 4.2. Blood Sample Extraction and Plasma Processing

Blood samples were extracted from subjects with CM [12] at any of three different time points during treatment: M0, M6 or M12.

Plasma samples were obtained in the early morning, between 9 am and 9:30 am, from the cubital vein using EDTA-K tubes at the University Hospital Marqués de Valdecilla. Blood samples were immediately centrifuged at 3500 rpm for 10 min to obtain plasma. Immediately after centrifugation, aliquots were prepared, with 10% of PI (Sigma-Aldrich, Burlington, MA, USA [65]) added to some, and stored at −80 °C until the day of testing. PI addition minimizes peptide degradation, ensuring that measured CGRP levels more accurately reflect true biological variation.

### 4.3. Laboratory and ELISA Procedures

α-CGRP and β-CGRP levels in plasma were measured using commercial ELISA tests, using Abbexa CGRP1 (CALCA) ELISA kits for α-CGRP (Abbexa Ltd., Cambridge, UK) [66] and Cusabio Human Calcitonin Gene Related Peptide ELISA kits for β-CGRP (Cusabio Biotech Co., Wuhan, China) [67], following the manufacturers’ instructions.

Briefly, standards and test samples were added and incubated in the ELISA plate, which was already pre-coated with the capture antibody, at 37 °C. After that, the biotin- conjugated reagent was added to the wells and incubated. After some washing steps, HRP-conjugated reagent was added and the plate incubated. Washing steps were again required and TMB substrate was used to quantify the HRP enzymatic reaction. After TMB addition, only wells with enough α-CGRP (for the Abbexa kit) and β-CGRP (for the CUSABIO kit) would produce a blue-colored product that would change to yellow after adding acidic stop solution. The intensity of yellow was proportional to the α-CGRP/β-CGRP amount bound on the plate in each case. The optical density (OD) was measured spectrophotometrically at 450 nm in a microplate reader, from which the concentration of α-CGRP and β-CGRP could be calculated afterwards [66,67]. Concerning the last step of the incubation of the TMB substrate, in which the manufacturers leave an open range of time in both the Abbexa and Cusabio kits and do not specify the exact time of incubation, we incubated the TMB substrate for 10 min for α-CGRP and 20 min for β-CGRP.

Samples from the same individual with both standard plasma and PI-plasma were allocated to the dame ELISA plate in order to minimize inter-assay variation. All the plasma samples were measured in duplicate, and the results of the concentrations were obtained by generating a standard curve using a 4-parameter logistic regression, subtracting the minimum OD value (of blank and standards) from all other ODs. Arigo’s Elisa calculator [68] was used to calculate the concentrations of CGRP.

The Abbexa Human ELISA kit has been reported to have a sensitivity in the range of (≈1.9–9.4 pg/mL) and a detection range of (≈3–15 pg/mL up to 200–1000 pg/mL). The manufacturer describes the assay as highly sensitive and specific; however, detailed validation data such as cross-reactivity are not disclosed. The CUSABIO ELISA kit has been reported to have a sensitivity of 0.39 pg/mL and a detection range of 1.56–100 pg/mL. The manufacturer states that the assay detects β-CGRP specifically and does not cross-react with α-CGRP, although detailed cross-reactivity testing is not disclosed either.

### 4.4. Data and Statistical Analysis

Continuous variables were reported as median with 95% CI for non-normally distributed data, unless it is stated differently in the text.

The normality of the variables was checked using normality tests (D’Agostino and Pearson test, Shapiro–Wilk test, Kolmogorov–Smirnov test) as well as visually by using a QQplot in GraphPad.

Correlation relationships were evaluated by Spearman’s correlation. Statistical differences between groups for non-normally distributed data and independent groups were checked with the Mann–Whitney U test, whereas for non-normally distributed paired samples, the Wilcoxon Signed-Rank test was used.

For multiple group comparisons among the same individuals at different time points, the Wilcoxon matched-pairs Signed-Rank test was used when only M0 and M6 samples were available, whereas comparisons between M0, M6 and M12 samples were checked using the Friedman test followed by Dunn‘s test to study specifically between which time points there was a statistical change.

Statistical tests were performed using GraphPad Prism version 10 and the *p* values presented are for two-tailed testing, and a *p* < 0.050 was considered as proving statistical significance.

## 5. Conclusions

With these findings we can support the importance of sample processing conditions since the addition of PI to plasma samples did change the CGRP levels for a more accurate quantification of the peptide, especially for α-CGRP. The lack of correlation between the α- and β-CGRP levels in plasma supports the idea that both isoforms could be independently regulated and may undertake distinct biological roles in CM disease, supporting what has already been mentioned in the literature, in which the α-CGRP isoform relates more with CM and the nervous system whereas β-CGRP is more related with the gastrointestinal system [69]. The β-CGRP levels were higher in plasma than in serum, which encourages the use of this biofluid when this isoform should be measured. The consistent decline in α-CGRP with migraine treatment highlights its potential as a reliable biomarker for monitoring therapeutic response, although further studies should be carried out. Additionally, demographic factors like age could affect CGRP levels as was shown in our results; however, we did not find a statistical difference in sex as had been said in previous articles published in the literature [5], probably because we had a small cohort of men, as we know that migraine frequently affects a major number of women, and our results could not reflect a reliable result.

## Data Availability

The original contributions presented in this study are included in the article. Further inquiries can be directed to the corresponding author.

## Abbreviations

The following abbreviations are used in this manuscript:

α-CGRP	Alpha-CGRP
β-CGRP	Beta-CGRP
CGRP	Calcitonin gene-related peptide
TG	Trigeminal ganglion
CGRP-R	CGRP receptor
CI	Confidence interval
CM	Chronic migraine
ELISA	Enzyme-linked immunosorbent assay
M0	Samples collected at baseline
M6	Samples collected after 6 months of treatment
M12	Samples collected after 12 months of treatment
mAbs	Monoclonal antibodies
NDDs	Neurodegenerative diseases
No PI-plasma	Plasma samples without PI
Ns	Non-significant
PI	Protease inhibitors
PI-plasma	Plasma samples with PI
Rs	Spearman’s correlation coefficient
